# Pulmonary vein encirclement using an Ablation Index-guided point-by-point workflow: cardiovascular magnetic resonance assessment of left atrial scar formation

**DOI:** 10.1093/europace/euz226

**Published:** 2019-09-05

**Authors:** Louisa O’Neill, Rashed Karim, Rahul K Mukherjee, John Whitaker, Iain Sim, James Harrison, Orod Razeghi, Steven Niederer, Tevfik Ismail, Matthew Wright, Mark D O’Neill, Steven E Williams

**Affiliations:** 1 Division of Imaging Sciences and Biomedical Engineering, King’s College London, 4th Floor North Wing, St. Thomas’ Hospital, London SE1 7EH, UK; 2 Department of Cardiology, Guy’s and St Thomas’ NHS Foundation Trust, London SE1 7EH, UK

**Keywords:** Pulmonary vein isolation, Ablation Index, Point-by-point ablation, Cardiac magnetic resonance imaging, Atrial ablation scar

## Abstract

**Aims:**

A point-by-point workflow for pulmonary vein isolation (PVI) targeting pre-defined Ablation Index values (a composite of contact force, time, and power) and minimizing interlesion distance may optimize the creation of contiguous ablation lesions whilst minimizing scar formation. We aimed to compare ablation scar formation in patients undergoing PVI using this workflow to patients undergoing a continuous catheter drag workflow.

**Methods and results:**

Post-ablation cardiovascular magnetic resonance imaging was performed in patients undergoing 1st-time PVI using a parameter-guided point-by-point workflow (*n* = 26). Total left atrial scar burden and the width and continuity of the pulmonary vein encirclement were determined on analysis of atrial late gadolinium enhancement sequences. Comparison was made with a cohort of patients (*n* = 20) undergoing PVI using continuous drag lesions. Mean post-ablation scar burden and scar width were significantly lower in the point-by-point group than in the control group (6.6 ± 6.8% vs. 9.6 ± 5.0%, *P* = 0.03 and 7.9 ± 3.6 mm vs. 10.7 ± 2.3 mm, *P* = 0.003). More complete bilateral pulmonary vein encirclements were seen in the point-by-point group (*P* = 0.038). All patients achieved acute PVI.

**Conclusion:**

Pulmonary vein isolation using a point-by-point workflow is feasible and results in a lower scar burden and scar width with more complete pulmonary vein encirclements than a conventional drag lesion approach.

**What’s new?**

- Point-by-point workflows with targeted Ablation Index (AI) values focus on formation of contiguous, durable lesion sets and are associated with high single procedure success rates for pulmonary vein isolation (PVI).
- This study assesses atrial ablation scar formation using late gadolinium enhancement cardiac magnetic resonance imaging in patients undergoing an AI-guided workflow and compares to control patients undergoing PVI using a continuous drag workflow.
- Scar burden was significantly lower in the point-by-point patients driven by a reduction in the overall width of the pulmonary vein encirclement.
- More complete pulmonary vein encirclements were achieved in the point-by-point group.
- This study suggests that equally effective lesion sets may be achieved in the presence of a lower scar burden which may offer important clinical benefits to patients.

## Introduction

Despite ongoing technological advances in catheter ablation for atrial fibrillation (AF), single procedure success rates remain modest. Conducting gaps in the pulmonary vein encirclement are consistently identified as a cause of arrhythmia recurrence^1^ and ablation lesion transmurality and contiguity are major determinants of electrically conducting gaps.^2^^,^^3^ The Ablation Index (AI; Carto3, Biosense Webster) is a real-time lesion assessment index incorporating contact force (CF), time, and power in a weighted formula. Ablation Index has been shown in canine studies to predict lesion depth^4^ and in humans to identify sites of pulmonary vein reconnection at repeat procedure.^5^^,^^6^ An initial study of a point-by-point workflow with targeted AI values and interlesion distances demonstrated high single procedure success rates.^7^

Atrial late gadolinium enhancement cardiovascular magnetic resonance (LGE-CMR) is an established tool in the assessment of post-ablation atrial scar formation.^8^ In this study, we used LGE-CMR to evaluate left atrial scar created during pulmonary vein isolation (PVI) using a point-by-point workflow with targeted AI values and interlesion distances. We hypothesized that this workflow would result in lower left atrial scar burden with more contiguous lesions than a conventional drag lesion approach.

## Methods

Consecutive patients undergoing 1st-time PVI using a point-by-point workflow and post-procedure CMR were recruited between January and December 2017. The control group consisted of an historical cohort of consecutive patients who underwent PVI using drag lesions plus post-procedural CMR between 2014 and 2015.^9^ All cases were carried out by the same two operators across both study groups. Data were collected as part of routine clinical care. Ethical approval was granted for retrospective data analysis (REC number 18/HRA/0083).

### Ablation procedure

#### Ablation Index calibration

To calibrate target AI values, both operators performed 10 cases using the point-by-point strategy blinded to AI. The mean AI of all lesions was taken to represent the target AI. Resulting AI targets were 400 throughout for Operator 1 and 400 anteriorly and 350 posteriorly for Operator 2.

#### Parameter-guided, point-by-point ablation group

**Figure 1:**
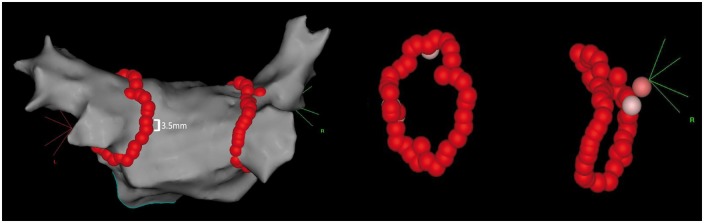
Ablation tag maps in the point-by-point lesion workflow. Left atrial anatomical map, shown in PA view demonstrating ablation lesions employed using a point-by-point workflow. Maximum interlesion distance was <6 mm, and Visitags were displayed once target AI values were achieved resulting in contiguous lesion sets around the pulmonary veins. Pink tags represent lesions with AI values <50 units below the target value. AI, Ablation Index; PA, posteroanterior.

Following induction of general anaesthesia, femoral venous access was obtained and a 6-Fr decapolar reference catheter was placed in the coronary sinus. Single trans-septal puncture was performed following which a 20-pole circular mapping catheter (Lasso; Biosense Webster Diamond Bar, CA, USA) and a 3.5-mm tip irrigated ablation catheter (Thermocool SmartTouch; Biosense Webster, Diamond Bar, CA, USA) were placed in the left atrium. Intravenous heparin was administered to maintain activated clotting time >300 s. Wide area circumferential PVI was performed using point-by-point ablation (*Figure 1*) in power control mode at 30 W (irrigation flow 17 mL/min). Radiofrequency applications were displayed using Visitag (Carto, Biosense Webster) with predefined catheter stability (2.5 mm for 3 s) and contact force (CF; >3 g for 25% of time) settings. Once target AI values were achieved the catheter was moved to the next site, accepting a maximum interlesion distance of 6 mm. If first pass isolation was not achieved, further ablation was performed guided by sites of earliest pulmonary vein activation. Isolation was reconfirmed at 30 min and further ablation delivered in the case of reconnection. Cavotricuspid isthmus ablation was performed after PVI in cases of documented typical atrial flutter.

#### Control group

Femoral access, trans-septal puncture, and catheter placement were as described above. Wide area circumferential ablation was performed using a CF sensing 3.5mm irrigated catheter (Thermocool SmartTouch, Biosense Webster). Continuous drag lesions were created in a temperature-controlled mode with power fixed to 25 W throughout for Operator 1 and 30 W at the anterior wall and 25 W at the posterior wall for Operator 2 with catheter movement every 20–30 s. Pulmonary vein isolation was confirmed as above.

### Atrial cardiovascular magnetic resonance acquisition

Cardiovascular magnetic resonance imaging was performed at 3 months post-procedure on a 1.5 T CMR scanner (Ingenia; Philips Medical Systems or Magnetom Aera; Siemens). Respiratory gated, electrocardiogram (ECG)-triggered magnetic resonance angiography (MRA) was performed 90 s post-infusion of 0.2 mmol/kg gadobutrol (Gadovist, Bayer Healthcare Pharmaceuticals, Berlin, Germany) with coverage to include the whole of the left atrium in axial orientation.

Twenty minutes after contrast administration three-dimensional LGE imaging was performed using an ECG-triggered, respiratory navigated inversion recovery sequence^9^ acquired in axial orientation using the same scan parameters as the MRA. A preceding single slice, multi-phase inversion time mapping sequence (Look Locker or TI-Scout) was used to determine the correct inversion time to achieve adequate nulling of ventricular myocardium. Spatial resolution was 1.29 × 1.29 × 4 mm reconstructed to 0.94 × 0.94 × 2 mm [repetition time (TR) 4 ms, echo time (TE) 2 ms, flip angle 20°] for Philips sequences and 1.3 × 1.3 × 4 mm reconstructed to 1.3 × 1.3 × 2 mm (TR 4 ms, TE 2 ms, flip angle 20°) for Siemens sequences.

### Atrial cardiovascular magnetic resonance image processing

#### Calculation of total ablation scar burden

All LGE sequences were processed according to previously described methods.^9^^,^^10^ A semi-automatic segmentation of the left atrial blood pool was created from the MRA following which an atrial surface shell was created. The pulmonary veins and left atrial appendage were removed and, using an intensity projection technique, the maximum signal intensity within 3 mm of the endocardial border was displayed on the surface of the shell. To evaluate total scar burden, a threshold of 3.3 SD above the mean blood pool signal intensity was employed which we have previously validated against histology in a porcine model of atrial ablation.^11^ Total post-ablation scar burden was calculated as the percentage of the atrial shell with a signal intensity above this threshold.

#### Analysis of width and continuity of pulmonary vein encirclement

**Figure 2:**
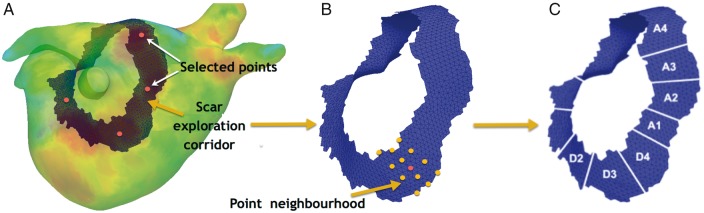
CMR analysis of the pulmonary vein encirclement. A path was drawn around each pulmonary vein encirclement (*A*) and signal intensities analysed within a 5-mm corridor (*B*). In order to characterize scar width and continuity, the encirclement was subdivided into equal sectors and signal intensities and spatial location of high signal intensity points analysed per sector (*C*). CMR, cardiovascular magnetic resonance.

Scar width was determined as follows. Points around both pulmonary vein encirclements were manually selected and the path between consecutive points computed using a shortest path graph algorithm^12^ allowing the reconstruction of the total encircled path around each pulmonary vein pair.^10^ The path was widened to allow exploration of gaps within a 5 mm corridor around each vein (*Figure 2*). For calculation of scar width, each encirclement was subdivided into 100 equal sectors. Within each sector, the signal intensity and spatial location of high signal intensity points were determined. Scar width was defined as the distance between two maximally distant points, above the scar threshold, perpendicular to the ablation path. To assess the continuity of the lesion set, the encirclement was divided into 16 equal sectors and signal intensity points analysed. A complete encirclement was defined as one in which the mean signal intensity in each of the 16 sectors was above the scar threshold.

### Statistical analysis

Data analysis was performed using SPSS statistics (IBM, Version 24) and Prism (GraphPad Software, Version 7). Normally distributed continuous variables were expressed as mean ± standard deviation. Comparison of means between groups was performed using independent samples *T*-test for normally distributed data and Mann-Whitney *U*-test for non-uniformly distributed data. *P*-value <0.05 was considered statistically significant.

## Results

### Baseline demographics

26 patients (13 male, 88% paroxysmal AF) were recruited to the point-by-point group and 20 patients (13 male, 90% paroxysmal AF) to the control group. There were no statistically significant differences in baseline demographics between the two study groups (*Table 1*). Acute PVI was achieved in all patients.

**Table 1:** Baseline patient demographics and characteristics

	Point-by-point	Control	*P*-value
Age (years)	61.7 ± 9.7	60.7 ± 11.4	0.947
Male, *n* (%)	13 (50)	13 (65)	0.309
Paroxysmal AF, *n* (%)	23 (88)	18 (90)	0.868
AF duration (years)	6.9 ± 8.4	4.1 ± 5.0	0.473
Hypertension, *n* (%)	12 (46.1)	6 (30)	0.179
Diabetes, *n* (%)	3 (12)	2 (10)	0.458
CHA_2_DS_2_VASc ≥ 2, *n* (%)	13 (50)	10 (50)	1.0
Anti-arrhythmic drugs, *n* (%)	10 (38)	9 (45)	0.655

AF, atrial fibrillation.

### Cardiovascular magnetic resonance analysis

**Figure 3:**
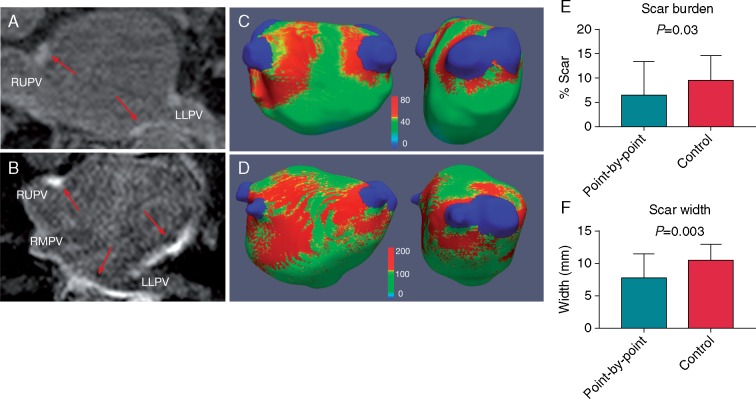
LGE-CMR images and scar maps. *Left panel*: LGE-CMR imaging. Red arrows indicate sites of high signal intensity surrounding the pulmonary veins and in the left atrial body in a point-by-point (*A*) and control patient (*B*). *Middle panel*: corresponding three-dimensional left atrial scar maps, thresholded at 3.3 SD above the mean blood pool SI demonstrate a lesser degree of scar (red areas), with a narrower encirclement width in point-by-point (*C*) vs. control patients (*D*). Colour scale represents raw signal intensity. Total scar burden (*E*) and scar width (*F*) were significantly lower in point-by-point vs. control groups CMR, cardiovascular magnetic resonance; LGE, late gadolinium enhancement; LLPV, left lower pulmonary vein; RMPV, right middle pulmonary vein; RUPV, right upper pulmonary vein; SI, signal intensity.

**Figure 4:**
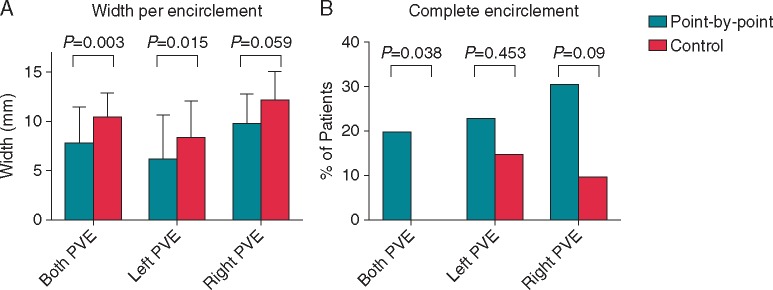
Scar width and gaps per encirclement. Scar width was lower across both encirclements for the point-by-point workflow, reaching statistical significance on the left (*A*). More complete encirclements were seen in the point-by-point group than in the control group (*B*). PVE, pulmonary vein encirclement.

Mean time to CMR was 3.6 ± 1.0 months for the point-by-point group and 3.6 ± 0.6 months for the control group (*P* = 0.903). Mean post-ablation total scar burden was significantly lower in the point-by-point group than in the control group (*P* = 0.03, *Table 2*, *Figure 3*). Scar width was significantly lower in the point-by-point group than in control group (*P* = 0.003, *Table 2*, *Figure 3*), driven by a significant difference in scar width on the left-sided encirclement (*P* = 0.015, *Table 2*, *Figure 4*). Similarly, scar width was lower on the right-sided encirclement in the point-by-point group but this did not reach statistical significance (*P* = 0.059, *Table 2*, *Figure 4*). Complete bilateral pulmonary vein encirclement, as defined by CMR, was seen in 5 (19.2%) point-by-point patients but was not seen in any of the control patients (*P* = 0.038, *Table 2*, *Figure 4*).

**Table 2:** CMR parameters

	Point-by-point	Control	*P*-value
Scar burden (%)	6.6 ± 6.8	9.6 ± 5.0	0.03
Total scar width (mm)	7.9 ± 3.6	10.7 ± 2.3	0.003
Left PVE scar width (mm)	6.4 ± 2.9	8.5 ± 2.8	0.015
Right PVE scar width (mm)	9.9 ± 4.4	12.3 ± 3.6	0.059
Complete bilateral PVE, *n* (%)	5 (19)	0	0.038
Complete left PVE, *n* (%)	6 (23)	3 (15)	0.453
Complete right PVE, *n* (%)	8 (31)	2 (10)	0.09

CMR, cardiovascular magnetic resonance; PVE, pulmonary vein encirclement.

### Procedural data and follow-up

Both procedure and fluoroscopy times were significantly lower in the point-by-point group than in the control group (147.8 ± 30.3 vs. 186.1 ± 45.3 min, *P* = 0.001 and 3.6 ± 2.5 vs. 9.2 ± 10.1 min, *P* = 0.006). Although radiofrequency (RF) time was lower in the point-by-point group, this was not statistically significant (35.3 ± 9.8 min vs. 41.6 ± 15.3 min, *P* = 0.101). There were no significant procedural complications in either group. One patient in the point-by-point group developed a groin haematoma. A further patient in the point-by-point group had an asymptomatic 50% narrowing documented in one pulmonary vein on post-ablation CMR. At 1 year follow-up, 21 of 26 patients (81%) in the point-by-point group and 14 of 20 patients (70%) in the control group were free from arrhythmia recurrence (*P* = 0.396).

## Discussion

In this study, we have shown that PVI using a point-by-point workflow results in significantly less post-ablation left atrial scar formation with a narrower scar width and a higher proportion of complete pulmonary vein encirclements compared to a conventional ablation catheter drag approach.

### ‘Ablation Index’ guided point-by-point work flow

Despite ongoing technological advances in catheter ablation, there has been limited improvement in the success rates of PVI. With AF recurrences known to be attributable to pulmonary vein reconnection recent attention has turned to intra-procedural characterization of gaps^13^ and ablation techniques aimed at reducing rates of long-term pulmonary vein reconnection.

The introduction of CF sensing catheters and the force-time integral (FTI) has improved gap prediction and outcomes post-PVI^14^ but does not take into account energy delivery to the atrial myocardium. Furthermore, in a porcine model of atrial ablation, no difference in lesion size was seen between lesions created using high and low CF.^15^ The Ablation Index incorporates power, force and time in a weighted formula and has been shown to be predictive of lesion depth and sites of pulmonary vein reconnection.^5^ Lesion contiguity is also crucial to vein reconnection^3^ with significantly higher interlesion distances previously described in reconnected segments of the pulmonary vein encirclement.^6^ High single procedure success rates have been reported using the CLOSE protocol, a point-by-point, AI-guided workflow,^16^ and the protocol employed in our study was similarly focused on the creation of contiguous lesions using a point-by-point work flow with a maximum interlesion distance of 6 mm. Operator-specific AI targets were determined based on average values obtained to achieve acute PVI in a series of operator-blinded cases.

### Cardiovascular magnetic resonance analysis

The central finding of this study is a reduction in post-ablation left atrial scar burden with point-by-point ablation, due to an overall reduction in scar width. A narrower scar width may be explained by reduced catheter movement consequent on enhanced catheter stability targets. Stable RF delivery, as a result of strict adherence to catheter stability and AI targets, has been attributed to improved outcomes in AI-guided point-by-point workflows^17^ and may explain the narrower scar width seen in the point-by-point group in this study. Further supporting these findings, in a bovine model, Olson *et al.*^18^ report a higher lesion volume, a function of both depth and width, in an ablation line created using drag vs. point lesions. Prior studies of post-ablation LGE-CMR reported lower recurrence rates in patients with more circumferentially scarred veins and higher total left atrial scar burden.^19^ In contrast, in this study, similar acute procedural success rates were achieved in both groups suggesting that acute PVI can be achieved, using this protocol, in the presence of lower scar burden. Furthermore, similar one-year success rates suggest that this type of lesion set remains durable in the medium term.

Conflicting results exist regarding the relationship between CMR-defined gaps and sites of electrical reconnection at repeat procedure.^20^^,^^21^ In this study, we did not attempt to relate the location of CMR defined gaps with electrically conducting gaps but rather sought to characterize the appearances of pulmonary vein scar formation in terms of scar morphology. We noted a greater proportion of complete bilateral pulmonary vein encirclements in the point-by-point group vs. the control group, further supporting the idea that the integrity of a lesion set is not dependent on total scar burden but rather depends on scar morphology.

### Clinical implications

The workflow employed in this study differs from other current strategies using a point-by-point workflow. By accounting for the contribution of power on lesion formation, the AI can potentially overcome some of the difficulties associated with CF or FTI-guided ablation. Similar AI values are expected to produce similar lesion depths irrespective of CF employed^22^ therefore, sufficient lesion quality can be expected even in areas where CF is low. Similarly, high power delivery may allow shorter duration applications and greater procedural efficiency with a reduction in procedure times.^7^

A reduction in left atrial compliance can occur post-catheter ablation with an association demonstrated between left atrial dysfunction and scar burden on LGE-CMR.^23^ Furthermore, reduced atrial contractility may have implications for thrombogenesis with a relationship previously described between absent LA contraction and stroke in post-surgical MAZE patients.^24^ Finally, excessive left atrial ablation is associated with damage to extracardiac structures.^25^ This suggests that a lower ablation scar burden may be advantageous. As such the ablation approach described here, by minimizing ablation whilst delivering comparable acute outcomes, may have important associated clinical benefits. Further studies are needed to fully assess this point.

### Limitations

The use of a cohort of historical controls may be responsible for an element of chronology and performance bias, and greater operator experience at time of the point-by-point procedures should be considered when interpreting the results presented. Power settings in the control group were at the discretion of the operators, and correspondingly target AI values varied between operators in the point-by-point group after the initial calibration phase. These differences may have resulted in differences in scar formation between operators not assessed for in this study. Furthermore, the lower power settings used by Operator 1 in the control group may have contributed to the lower proportion of complete bilateral encirclements in this group. The proportion of complete bilateral encirclements is low in this study. It is worth noting that the amount of tissue classified as ablation scar is dependent on the CMR thresholding technique utilized. The threshold employed in this study has previously undergone histological validation, however, it is likely that had a lower scar threshold been chosen a greater proportion of complete encirclements would have been seen. As such the absolute values presented are of less relevance than the relative differences seen between groups using the same image processing methods.

Although 1-year outcome data are presented, this was an observational single centre study not powered to assess for differences in arrhythmia recurrence.

## Conclusion

Pulmonary vein isolation using a point-by-point, AI-guided workflow results in a lower overall scar burden and narrower scar width than a drag lesion approach. More CMR defined complete pulmonary vein encirclements were observed with the proposed point-by-point workflow.
